# Human prostate cell lines from normal and tumourigenic epithelia differ in the pattern and control of choline lipid headgroups released into the medium on stimulation of protein kinase C

**DOI:** 10.1038/sj.bjc.6606077

**Published:** 2011-01-25

**Authors:** M Rumsby, J Schmitt, M Sharrard, G Rodrigues, M Stower, N Maitland

**Affiliations:** 1 Department of Biology, University of York, York YO10 5DD, UK; 2 Department of Pathology, Hull Royal Infirmary, Hull HU3 2JZ, UK; 3 Department of Urology, York District Hospital, York YO31 8HE, UK

**Keywords:** prostate epithelial cell lines, protein kinase C, phospholipase D, choline and phosphocholine release, MARCKS

## Abstract

**Background:**

Expression of protein kinase C alpha (PKC*α*) is elevated in prostate cancer (PCa); thus, we have studied whether the development of tumourigenesis in prostate epithelial cell lines modifies the normal pattern of choline (Cho) metabolite release on PKC activation.

**Methods:**

Normal and tumourigenic human prostate epithelial cell lines were incubated with [^3^H]-Cho to label choline phospholipids. Protein kinase C was activated with phorbol ester and blocked with inhibitors. Choline metabolites were resolved by ion-exchange chromatography. Phospholipase D (PLD) activity was measured by transphosphatidylation. Protein expression was detected by western blotting and/or RT–PCR. Choline uptake was measured on cells in monolayers over 60 min.

**Results:**

Normal prostate epithelial cell lines principally released phosphocholine (PCho) in contrast to tumourigenic lines, which released Cho. In addition, only with normal cell lines did PKC activation stimulate Cho metabolite release. Protein kinase C alpha expression varied between normal and tumourigenic cell lines but all showed a PKC*α* link to myristoylated alanine-rich C kinase substrate (MARCKS) protein. The five cell lines differed in Cho uptake levels, with normal PNT2C2 line cells showing highest uptake over 60 min incubation. Normal and tumourigenic cell lines expressed mRNA for PLD1 and PLD2, and showed similar levels of basal and PKC-activated PLD activity.

**Conclusions:**

The transition to tumourigenesis in prostate epithelial cell lines results in major changes to Cho metabolite release into the medium and PKC signalling to phosphatidylcholine turnover. The changes, which reflect the metabolic and proliferative needs of tumourigenic cells compared with untransformed cells, could be significant for both diagnosis and treatment.

Conventional protein kinase C alpha (PKC*α*) is linked to the regulation of cell proliferation, motility, survival, apoptosis, differentiation, metastasis and multidrug resistance (Gutcher *et al*, 2003; Gavrielides *et al*, 2004; Koivunen *et al*, 2006; Larsson, 2006). Protein kinase C alpha expression is reduced in many cancers (Koivunen *et al*, 2006; Griner and Kazanietz, 2007; Mackay and Twelves, 2007; Ali *et al*, 2009). However, early prostate adenocarcinomas (PCa) show increased PKC*α* protein (Cornford *et al*, 1999; Koren *et al*, 2004; Lahn *et al*, 2004). Rat prostatic tumour cell lines also show elevated PKC*α* expression over controls (Powell *et al*, 1994). Androgen-independent human prostatic epithelial carcinoma lines PC3 and DU145 express PKC*α* mRNA and protein more prominently than does the androgen-sensitive LNCaP line (Krongrad and Bai, 1994; Powell *et al*, 1996), although only LNCaP cells undergo PKC*α*-mediated apoptosis when stimulated with phorbol esters (O’Brian 1998; Gutcher *et al*, 2003; Gonzalez-Guerrico *et al*, 2005). However, PKC*α* mediates the apoptosis induced by activation of Toll-like receptor 3 in both LNCaP and PC3 lines (Paone *et al*, 2008). Protein kinase C alpha can affect the growth-inhibiting effects of transforming growth factor-*β* in PC3 cells (Lamm *et al*, 1997), as well as epidermal growth factor receptor transactivation and activation of Erk1/2 (Stewart and O’Brian, 2005). Protein kinase C alpha is a proposed therapeutic target in androgen-independent PCa (O’Brian, 1998); therefore, it is important to understand how elevated PKC*α* expression as observed in PCa influences downstream targets which are also implicated in tumourigenesis; for example, phospholipase D (PLD) (Cockcroft, 2001; Foster, 2009).

Phospholipase D expression and activity are elevated in several human tumours and neoplastic cell lines (Foster and Xu, 2003; Foster 2006, 2009), resulting in increased cell proliferation and prevention of cell-cycle arrest and apoptosis (Joseph *et al*, 2002; Zhong *et al*, 2003; Foster, 2006). These effects occur partly through the increased formation of phosphatidic acid (PtdOH), which modulates the activity of Raf and mammalian target of rapamycin (mTOR), both regulators of cell proliferation (Guertin and Sabatini, 2007; Foster, 2007a, 2007b). Mammalian target of rapamycin is also implicated in signals that suppress apoptosis in cancer cells enabling their survival and proliferation under stress conditions (Foster, 2009). Enhanced PLD activity results in elevated levels of Cho metabolites, especially phosphocholine (PCho), in breast and prostate cancer cells (Ackerstaff *et al*, 2001; Glunde *et al*, 2006) where PCho levels may be a useful biomarker of malignant disease (Eliyahu *et al*, 2007; Beloueche-Babari *et al*, 2009). Choline kinase (CK) activity is increased in many tumours (Ramirez de Molina *et al*, 2002; Gallego-Ortega *et al*, 2009) and is a link to cell-cycle regulation (Ramirez de Molina *et al*, 2008) through MAPK and PI3K/AKT signalling (Chua *et al*, 2009; de Souza *et al*, 2009; Yalcin *et al*, 2010).

Stimulation of phosphatidylcholine (PtdCho) turnover in cells (Kiss, 1990) results in the release of Cho metabolites into the medium (Mufson *et al*, 1981; Hii *et al*, 1991; van Blitterswijk *et al*, 1991; Troyer *et al*, 1992; Morreale *et al*, 1997) wherein PCho can promote the mitogenic activity of insulin and growth factors (Cuadrado *et al*, 1993; Tomono *et al*, 1995; Chung *et al*, 1997). In this study, we show that tumourigenesis in human prostate epithelial cell lines alters the nature and control of Cho metabolites released into the medium on PKC activation.

## Materials and methods

### Cell culture

PNT1A, PNT2C2 and LNCaP prostate epithelial cell lines (up to passages 80, 150 and 50, respectively) were cultured in RPMI1640 (Gibco, Invitrogen Ltd, Paisley, Scotland, UK) with 10 mM HEPES, 2 mM glutamine and 10% fetal bovine serum (FBS) (R10). PC3 cells up to passage 50 were grown in Ham's F12 medium (Lonza, Slough, Berkshire, UK) with 7% FBS (F7). P4E6 line cells (Maitland *et al*, 2001) were cultured in KSFM medium (Gibco) with epidermal growth factor and pituitary additives+2% FBS (K2). For passage/experimentation, cells were rinsed with Tris-saline (TS) and released with Tris-trypsin (TT) for 10 min. Trypsin was inactivated with R10, cells pelleted by centrifugation and resuspended in normal growth medium for counting.

### [^3^H]-Choline headgroup release into the medium

A total of 7.5 × 10^4^ cells were seeded in triplicate into wells of 24-well plates in normal growth medium (see above) and cultured overnight. For LNCaP cells, wells were coated with poly-L-lysine (20 *μ*g ml^−1^ in water) to aid adhesion. At 80–90% confluency, the medium was replaced with RPMI1640, F12 or KSFM containing 1% FBS and 0.5 *μ*Ci [^3^H]-choline (Perkin-Elmer, Beaconsfield, Buckinghamshire, UK) per well for 30 h to label Cho phospholipids to equilibrium. With this low level of serum, cells were just becoming confluent when used, and thus significant changes to enzyme activity because of contact inhibition or cell-cycle effects were avoided. Radioactive medium was then removed and cells were incubated for 60 min at 37°C with serum-free medium. Cells were gently rinsed twice more with warm (37°C) serum-free medium and 0.5 ml serum-free medium containing 1 mM choline chloride and 1 mM phosphocholine plus PKC activators/inhibitors (see Figure legends) was added per well. Aliquots (25 *μ*l) of medium were removed from wells at T=0 and then as appropriate. A volume of 25 *μ*l fresh medium was added back to wells to maintain volume. Aliquots of medium were centrifuged at 13 000 r.p.m. to pellet any cell debris. Duplicate 10 *μ*l aliquots were added to 96-well Top Count plates for scintillation counting with 75 *μ*l Microscint-20 (Perkin-Elmer Ltd.). Mean c.p.m. values from triplicate wells were calculated with s.d. (*n*=6). In experiments to reduce PKC*α* protein content, cells in triplicate wells were chronically treated with 250 nM TPA for the last 9 h of [^3^H]-choline labelling and then used as above.

### Vesicle release

A volume of 400 *μ*l of culture medium was removed from wells at the end of 3 h Cho release assays and triplicate 10 *μ*l aliquots were taken for Top-Count scintillation counting as above. The remaining medium was transferred to a Beckman Eppendorf tube and centrifuged for 5 min at 13 000 r.p.m. to sediment any cell debris. Again, triplicate 10 *μ*l aliquots of the low-speed supernatant were removed for counting. The remaining medium was then centrifuged at 100 000 g for 30 min at 4 °C to sediment exosomes and/or other vesicles (Nilsson *et al*, 2009). Aliquots (10 *μ*l) of supernatant medium from this step were also counted.

### Distribution of [^3^H]-Cho in intracellular Cho metabolites and lipids on labelling

Cells were seeded into wells of 24-well plates in triplicate and labelled with 0.5 *μ*Ci [^3^H]-Cho for 30 h as above. The labelling medium was removed and cells were rinsed three times with ice-cold PBS before extraction with 0.5 ml methanol, 0.5 ml chloroform:methanol, (1 : 2 v/v) and twice with 0.5 ml chloroform : methanol (1 : 1v/v). Solvent extracts were pooled, chloroform added to a final ratio of chloroform : methanol 2 : 1 and solvent/aqueous phases separated by the addition of 0.1 M KCl. Aliquots of each phase were taken in triplicate for scintillation counting. Choline metabolites in 400 *μ*l top phase or medium from release experiments were diluted to 5 ml in distilled water and added to Dowex-50WH+ ion exchange resin columns to resolve GPCho (glycerylphosphorylcholine), PCho and Cho (Cook and Wakelam, 1989; Kiss *et al*, 1994). Radioactivity in triplicate 0.5 ml aliquots of each fraction was measured by scintillation counting to calculate total dpm/fraction.

### Western blotting

A total of 1 × 10^5^ cells were seeded into 24-well plates in normal growth medium and cultured overnight. The medium was removed and cells were rinsed with Tris/saline and solubilised in 100 *μ*l warm (37°C) 2 × Laemmli sample buffer (Sigma-Aldrich, Poole, Dorset, UK) containing protease and phosphatase inhibitor pellets (Roche Diagnostics Ltd, Burgess Hill, West Sussex, UK). Extracts were heated at 100°C for 10 min. Equal volumes of cell extracts were resolved by SDS–PAGE on 12.5% gels for western blotting (Dawson *et al*, 2003) on Immobilon P (Millipore, Dundee, Scotland, UK). Protein kinase C alpha, actin and GAPDH blots were blocked in 5% Marvel/Tris buffered saline-0.2% Tween 20 (TBST). Phospho-MARCKS blots were blocked in 4% bovine serum albumin (BSA)/TBST. A mouse monoclonal antibody against the *C*-terminal V5 region of PKC*α* was prepared by Professor Nigel Groome (Oxford Brookes University) and used at 1 : 100. A MARCKS phospho (pS159/163) rabbit monoclonal antibody (Epitomics, InSight Biotechnology Ltd, Wembley, Middlesex, UK) was used at 1 : 1000. A polyclonal anti-actin antibody (Sigma-Aldrich) was used at 1 : 1000. Polyclonal antibodies to PKC*δ* and PKC*ε* (Cell Signaling, New England Biolabs, Hitchin, Hertfordshire, UK) were used at 1 : 1000. An anti-GAPDH antibody (Abcam, Cambridge, Cambridgeshire, UK) was used at 1 : 2000. Detection was by ECL, and X-ray film was pre-flashed for densitometry using Image J. Protein kinase C alpha and p-MARCKS blots were stripped and reprobed for GAPDH or actin as loading controls.

### Reverse transcriptase–PCR

Cells were grown in 75 cm^2^ flasks, rinsed and total RNA was extracted using a Qiagen RNeasy mini kit (Qiagen, Crawley, West Sussex, UK) and QIA shredder. RNA was quantified spectrophotometrically and 1 *μ*g taken for cDNA synthesis using the Invitrogen SuperScript II RT (Invitrogen Ltd, Paisley, Scotland, UK) protocol. This was used to prepare a master mix with appropriate water controls for PCR. Conditions for amplification were 94°C for 0.5 min, 54°C for 0.5 min, 72°C for 1 min for 35 cycles. Primers were from Eurogentec Ltd (Southampton, UK). Sequences for hPLD1 and hPLD2 primers were as described by Gibbs and Meier (2000). Primers for PKC*α* and actin were as described by Myklebust *et al* (2000).

### Transphosphatidylation

A total of 5 × 10^5^ cells per well were cultured in duplicate in six-well plates in 2 ml normal growth medium to near confluency as mentioned above. Cells were rinsed and labelled for 6 h with 1 *μ*Ci [^3^H]-myristic acid (Amersham, GE Health Care, Chalfont St Giles, Buckinghamshire, UK) in 1 ml serum-free medium. Cells were then incubated for 30 min in fresh serum-free medium, which was then replaced with 1 ml fresh serum-free medium containing 0.3% *n*-butanol (Morris *et al*, 1997) and either 1 *μ*M 4*α*-phorbol, 1 *μ*M 12-*O*-tetradecanoylphorbol 13-acetate (TPA) or 1 *μ*M TPA+1 *μ*M Ro31-8220. Cells were incubated for 30 min at 37 °C, rinsed and lipids recovered with 1 ml methanol, followed by 1 ml each of 1 : 2 chloroform : methanol and 1 : 1 chloroform : methanol. Chloroform was added to combined extracts and a two-phase system was generated with 0.1 M KCl. The chloroform phase was evaporated, redissolved in 200 *μ*l C/M (2 : 1v/v) and triplicate 10 *μ*l aliquots were taken into scintillation vials for total counts. Triplicate 25 *μ*l aliquots were applied to oxalate-impregnated silicic acid TLC plates and overlayed with authentic PtdBut, PtdOH and PtdCho standards (Lipid Products, Nutfield, UK). Plates were developed in chloroform : methanol : acetic acid (9 : 1 : 1, v/v) and lipids detected with iodine. After removal of iodine, PtdBut, PtdOH and PtdCho adsorbent areas were scraped into vials for scintillation counting. Means of triplicate values were calculated, and dpm in PtdBut expressed as a percentage of PtdCho d.p.m.

### Myristoylated alanine-rich C kinase substrate phosphorylation

Cells seeded into 24-well plates as above were cultured overnight in normal growth medium before transfer into RPMI1640, F12 or KSFM containing 1% FBS for 24 h. At 9 h before experiments, some wells were treated with 250 nM TPA to downregulate PKC*α*. Subsequently, cells were rinsed with serum-free medium for 60 min and then stimulated for 0, 15 and 30 min with 1 *μ*M TPA. Cells were rinsed and immediately solubilised in warm 2 × Laemmli sample buffer (Sigma-Aldrich) containing protease and phosphatase inhibitors (Roche) for western blotting as above.

### Choline uptake

A total of 1 × 10^5^ cells were seeded with six replicates into BD amine (BD Biosciences, Oxford, UK) 24-well plates in their normal growth medium and cultured overnight. Cells were rinsed with serum-free medium and incubated for 60 min in the same medium. Cells were then rinsed once with TS and twice with Cho-free uptake buffer (Muller *et al*, 2009) at 37°C. This was finally replaced with 250 *μ*l uptake buffer containing 10 *μ*M Cho plus 1 *μ*Ci [^3^H]-Cho per well. Cells were incubated for 60 min at 37°C and uptake stopped by addition of 750 *μ*l ice-cold phosphate-buffered saline (PBS). Cells were rinsed three times with ice-cold PBS, solubilised in 250 *μ*l 1% SDS/0.2 M NaOH (Wang *et al*, 2007) and triplicate 25 *μ*l aliquots taken for scintillation counting. Use of BD amine plates ensured that LNCaP cells remained attached during rinsing.

### Statistical treatment

Statistical significance was determined by the Student's two-tailed *t*-test or by a one-way Anova and the Tukey HSD test.

## Results

### Protein kinase C alpha expression by prostate epithelial cell lines

**Figure 1:**
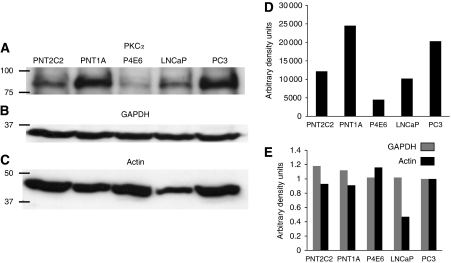
(**A**) Western blot detection of PKC*α* protein expression in PNT2C2, PNT1A, P4E6, LNCaP and PC3 prostate epithelial cell lines as described in Materials and Methods. The PKC*α* blot shown was stripped and reprobed for (**B**) GAPDH or (**C**) actin. Bands were quantified using Image J. (**D**) PKC*α* protein content of prostate epithelial cell lines normalised to GAPDH protein. (**E**) Comparison of GAPDH and actin protein content of prostate cell lines showing low actin content of LNCaP cells. Blots shown are typical of several repeats. Positions of 100, 75, 50 and 37 kDa markers are shown.

Reverse transcriptase–PCR with the same amount of total RNA from each cell line taken for reverse transcription confirmed that all lines express PKC*α* mRNA. The amplified band for PKC*α* was most prominent in PC3 cells and weakest in the P4E6 line (results not shown). Western blotting of equal cell numbers revealed that all lines express PKC*α* protein (Figure 1A). Expression of GAPDH protein by the five cell lines was almost uniform (Figure 1B and E). Actin expression was low in LNCaP cells compared with other lines (Figure 1C and E), making it an unsatisfactory loading control for comparison between the different cell lines used, although satisfactory for the same cell line. Bands in the PKC*α* blot were thus normalised to GAPDH (Figure 1D) to reveal differences in PKC*α* protein expression.

### Prostate cell lines express PKC*δ*, PKC*ε*, PKC*ζ* and MARCKS

Western blotting revealed that all five prostate cell lines express PKC*δ*, PKC*ε*, PKC*ζ* and MARCKS protein (results not shown).

### Myristoylated alanine-rich C kinase substrate phosphorylation

**Figure 2:**
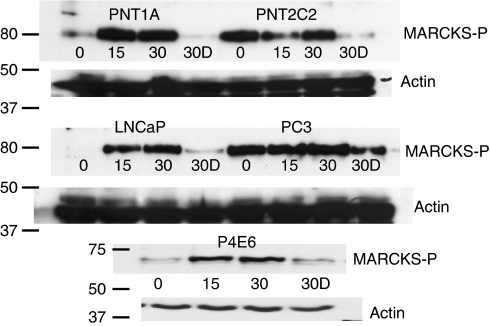
Phorbol ester stimulation of prostate cell lines results in MARCKS phosphorylation. Prostate cells were cultured, stimulated with TPA for 15 or 30 min (15, 30) and proteins resolved for western blotting to detect phospho-MARCKS as described above. Some cells were pretreated for 9 h with 250 nM TPA to downregulate PKC*α* protein before restimulation with 1 *μ*M TPA for 30 min (30D). In all lines, downregulation of PKC*α* protein results in significantly reduced MARCKS phosphorylation. Blots were stripped and reprobed for actin as a loading control. A repeat experiment gave similar results. Positions of 80, 75, 50 and 37 kDa markers are indicated.

TPA (1 *μ*M) stimulation of cells for 15 and 30 min increased MARCKS phosphorylation in PNT1A, P4E6 and LNCaP lines (Figure 2). Constitutively phosphorylated MARCKS was detected in PNT2C2 and PC3 cells, and this phosphorylation was not obviously increased by addition of TPA. Cells that had been exposed to 250 nM TPA for 9 h to downregulate PKC*α* showed almost complete suppression of MARCKS phosphorylation on restimulation with TPA, except for the PC3 line where MARCKS phosphorylation was at a reduced level (Figure 2).

### Prostate cell lines express mRNA for both PLD1 and PLD2

Reverse transcriptase–PCR with equal quantities of total RNA from each cell line taken for reverse transcription revealed that all five prostate epithelial cell lines express mRNA for PLD1 and PLD2 (results not shown). PLD1 mRNA expression was most prominent in PC3 cells. Phospholipase D2 mRNA expression was prominent in P4E6 and PC3 lines.

### Phospholipase D activity

**Figure 3:**
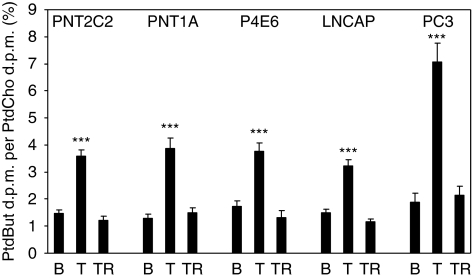
PNT2C2, PNT1A, P4E6, LNCaP and PC3 prostate cell lines show basal and TPA-stimulated PLD activity in the transphosphatidylation reaction. Cells were stimulated for 30 min with 4*α*-phorbol to measure basal (B) PLD activity, 1 *μ*M TPA (T) or 1 *μ*M TPA+1 *μ*M Ro31-8220 (TR). Lipids were extracted and resolved as described in Materials and Methods. Formation of PtdBut is expressed as % PtdBut d.p.m./PtdCho d.p.m. Results are ±s.e.m., *n*=3. ^***^P<0.0001 compared with basal and TPA/Ro31-8220-treated cells.

All cell lines showed basal (unstimulated) PtdBut formation (Figure 3) in the transphosphatidylation reaction indicating PLD activity. PtdBut formation was increased 2- to 2.5-fold when PNT2C2, PNT1A, P4E6 and LNCaP cell lines were treated with TPA to activate PKC, and by about three-fold in PC3 cells. In all cell lines, TPA-stimulated PtdBut formation was reduced to basal level by inclusion of the PKC inhibitor Ro31-8220 (Figure 3).

### [^3^H]-label is not released into the medium as vesicles or exosomes

**Figure 4:**
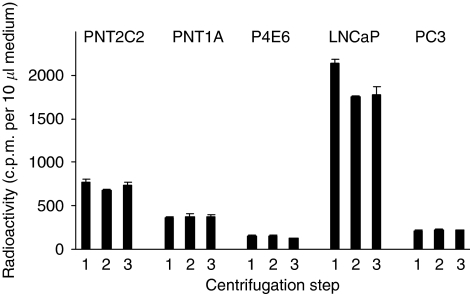
Prostate cell lines do not release [^3^H]-label into the medium as vesicles. Levels of radioactivity released into the medium from PNT2C2, PNT1A, P4E6 and PC3 cells did not decrease on low- or high-speed centrifugation as described. Results shown are for medium from cells stimulated with TPA for 3 h before centrifugation (1) and after low-speed (2) and high-speed (3) and are ±s.d., *n*=3.

Neither low- nor high-speed centrifugation (see Materials and Methods section) reduced levels of radioactivity in 3 h media from PNT2C2, PNT1A, P4E6 and PC3 lines (Figure 4). Radioactivity in medium from LNCaP cells decreased on low-speed centrifugation because of sedimentation of cells that had detached during the incubation. No further decrease in radioactivity occurred when LNCaP medium was centrifuged at 100 000 g (Figure 4).

### Only PNT2C2 and PNT1A line cells show a consistent phorbol ester-stimulated release of [^3^H]-label into the medium

**Figure 5:**
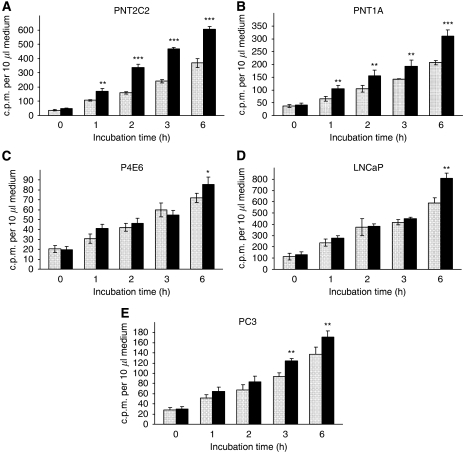
(**A–E**) Prostate epithelial cell lines show different patterns of [^3^H]-label release into the medium on stimulation with 1 *μ*M 4*α*-phorbol [ 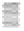 ] or 1 *μ*M TPA [ 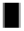 ] for up to 6 h as described in Materials and Methods. Results are means±s.d. (*n*=6). Repeat experiments (*n*=3) showed similar trends. For PNT2C2 and PNT1A lines ^**^P<0.001, ^***^P<0.0001 against basal values. For P4E6, LNCaP and PC3 lines ^*^P<0.05, ^**^P<0.01, ^***^P<0.001 against basal values.

**Figure 6:**
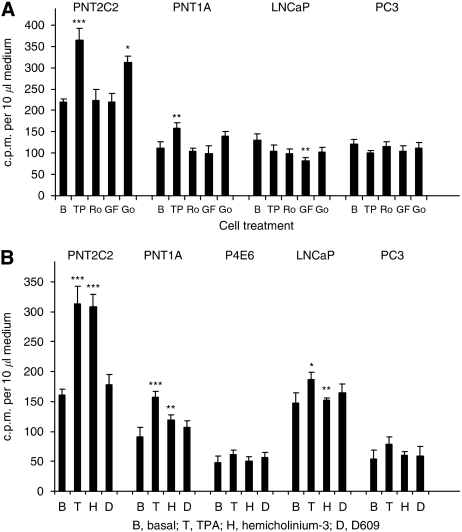
(**A**) Protein kinase C inhibitors reduce phorbol ester-stimulated release of [^3^H] label into the medium from PNT2C2 and PNT1A cells. PNT2C2, PNT1A, LNCaP and PC3 cell lines labelled with [^3^H]-choline were stimulated as described in Materials and Methods section with 1 *μ*M 4*α*-phorbol (basal, B), 1 *μ*M TPA (TP), 1 *μ*M TPA+1 *μ*M Ro31-8220 (Ro), 1 *μ*M TPA+1 *μ*M GF-109203X (GF) or 1 *μ*M TPA+1 *μ*M Go6976 (Go) and release of [^3^H]-label into the medium was monitored at 3 h. In both PNT2C2 and PNT1A cells, PKC inhibitors reduced release to basal values. Results are ±s.d. (*n*=6). For PNT2C2 and PNT1A cells, ^*^P<0.01, ^**^P<0.001, ^***^P<0.0001 against the basal value. For LNCaP cells ^**^P< 0.01 against the basal value. (**B**) D609 and hemicholinium-3 effects on [^3^H]-label release by prostate cell lines. PNT2C2, PNT1A, P4E6, LNCaP and PC3 cells labelled with [^3^H]-choline were stimulated as described in Materials and Methods with 1 *μ*M 4*α*-phorbol (basal, B), 1 *μ*M TPA (T), 1 *μ*M TPA+200 *μ*M HC-3 (H) or 1 *μ*M TPA+100 *μ*M D609 (D) and release of [^3^H]-choline metabolites to the medium was monitored at 3 h. Results are ±s.d. (*n*=6). For PNT2C2 cells ^***^P<0.0001 against the basal value. For PNT1A cells ^**^P<0.001 against the TPA value, ^***^P<0.0001 against basal value. For LNCaP ^*^P<0.05 against the basal value, ^**^P<0.01 against the TPA value.

Prostate cell lines labelled with [^3^H]-choline were stimulated with 4*α*-phorbol (basal) or TPA, and the medium collected to measure released radioactivity. Results of [^3^H]-label released in 6 h incubations are shown in Figure 5A–E and are typical of several repeats. In fact, Cho metabolite release by all cell lines increased linearly well beyond 6 h, but normal release experiments usually only extended to 3 h (Figure 6A and B) to avoid PKC downregulation caused by longer-term phorbol ester treatment. LNCaP cells were particularly difficult to work with in these experiments as they adhered poorly to plastic (even after polylysine treatment), leading to variable levels of Cho release (Figures 5D and 6A). A reproducible TPA-stimulated release of [^3^H]-label to the medium was only observed with PNT2C2 and PNT1A cell lines. This release effect was always greater in PNT2C2 cells than in the PNT1A line. Routinely, TPA had little or no stimulatory effect on the release of [^3^H]-label from P4E6 and LNCaP lines in incubations up to 3 h; however, by 6 h a significant stimulation of label release by TPA was observed, especially from LNCaP cells (Figure 5C and D). In the results shown in Figure 5E, for PC3 cells, TPA induced a small but significant release of [^3^H]-label to the medium at 3 and 6 h over basal. This was not a consistent effect, however, as shown by the results for TPA stimulation of PC3 cells in inhibitor experiments (Figure 6A and B). Dimethylsulphoxide (DMSO), solvent for phorbol esters and inhibitors, had no effect on [^3^H]-label release into the medium (results not shown). Levels of [^3^H]-label released into the medium by P4E6 and PC3 lines were consistently lower than levels released by PNT2C2, PNT1A and LNCaP lines, although equal cell numbers were seeded initially; cells were labelled identically and 60 min uptake rates (Figure 8) were the same as those in PNT1A cells. The three media used contained unlabelled choline chloride at 21 *μ*M (RPMI, KSFM) and 100 *μ*M (F12).

### Inhibition of phorbol ester-stimulated [^3^H]-radioactivity release

Ro31-8220 and GF109203X reduced TPA-stimulated release of [^3^H]-choline metabolites from PNT2C2 and PNT1A cells to basal values as shown in results from a 3-h incubation (Figure 6A). With LNCaP cells, GF109203X reduced [^3^H]-label release to the medium to below basal values. Go6976, a PKC inhibitor supposedly selective for *α* and *β*1 isoforms, had only a small inhibitory effect on [^3^H]-label release from PNT2C2 cells and was without significant effect on label release from PNT1A cells.

### Effects of hemicholinium-3 and D609

When monitored in a 3-h incubation (Figure 6B), D609 at 200 *μ*M almost completely inhibited the TPA-stimulated release of [^3^H]-label from PNT2C2 and PNT1A cells. The choline transporter inhibitor hemicholinium 3 (HC-3) at 100 *μ*M was without effect on [^3^H]-label released from PNT2C2 cells, and induced a partial but significant inhibition of label release from PNT1A cells. In the experiment shown (Figure 6B), activation of PKC with TPA stimulated a slight increase in label release from LNCaP cells, but this was not a consistent effect. Hemicholinium 3 reduced this stimulated release to basal levels. In this experiment, effects of TPA and inhibitors on PC3 cells were not significantly different from basal values.

### Nature of the choline metabolites released into the medium

**Figure 7:**
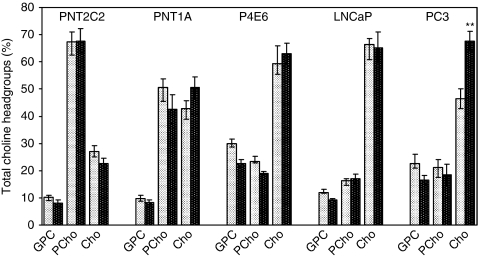
Tumourigenic prostate cell lines release mainly Cho into the medium as the principal metabolite, whereas PCho is released by non-tumourigenic PNT2C2 cells. GPC (GPCho), PCho and Cho in medium from 3 h time points from basal (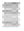) and TPA-treated (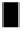) cells were fractionated as described in Materials and Methods section. Results for individual metabolites are expressed as percentage of the total GPCho+PCho+Cho d.p.m. measured±s.e.m. (*n*=4).

Medium from basal and phorbol ester-stimulated cells at 3 h time points was resolved into GPCho(GPC), PCho and Cho fractions on Dowex-50WH+ ion exchange resin columns (Figure 7). Results for each Cho metabolite are expressed as a % of the total Cho metabolites released (i.e., GPCho+PCho+Cho). Phosphocholine was the major metabolite released into the medium by basal and phorbol ester-stimulated PNT2C2 cells, whereas Cho was the major metabolite detected in media from basal and stimulated P4E6, LNCaP and PC3 lines. PNT1A cells were intermediate in that PCho and Cho each accounted for about equal proportions of the [^3^H]-label released. In the results shown for PC3 cells, Cho accounted for a higher proportion of the metabolites released on TPA stimulation (68%) compared with unstimulated cells (45%). With all other lines, the proportions of GPCho, PCho and Cho were the same in unstimulated and TPA-stimulated cells. Thus, in PNT2C2 cells, TPA treatment increased Cho metabolite release, but the proportions of GPCho : PCho :Cho were the same as from unstimulated cells.

### [^3^H]-Choline uptake by prostate cell lines

**Figure 8:**
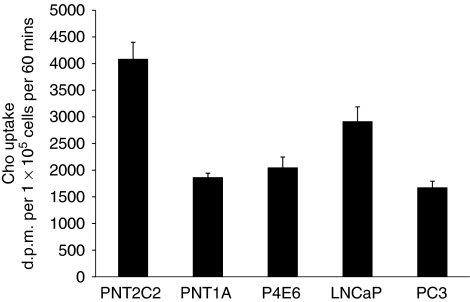
Comparison of Cho uptake by non-tumourigenic and tumourigenic prostate cell lines in monolayer culture. Results are shown as d.p.m. per 25 *μ*l from 1 × 10^5^ cells solubilised in 250 *μ*l over 60 min at 37°C as described in Materials and Methods section and are ±s.d. (*n*=6).

PNT2C2 cells at 1 × 10^5^ cells per well showed a greater uptake of [^3^H]-Cho label over 60 min at 37°C (Figure 8) compared with the other four cell lines where levels taken up were more comparable under identical conditions.

### [^3^H]-Choline distribution in Cho metabolites and phospholipids after labelling

**Figure 9:**
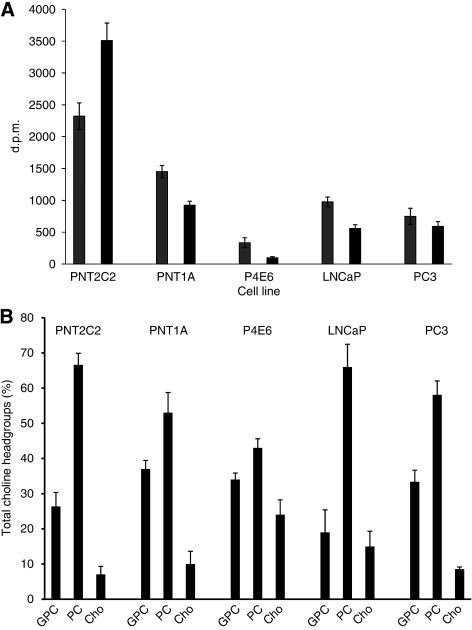
(**A**) Distribution of [^3^H]-Cho in lipid 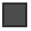 and aqueous 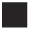 phases of prostate cell lines immediately after labelling cells as described in Materials and Materials and Materials and Methods. Results are d.p.m.±s.d. (*n*=3). (**B**) Distribution of [^3^H]-Cho label in GPC(GPCho), PCho and Cho in prostate epithelial cell lines immediately after labelling as described in Materials and Methods. Results for individual metabolites are expressed as percentage of the total GPC+PCho+Cho dpm±s.d. (*n*=3).

Over the 30-h labelling period, PNT1A, P4E6, LNCaP and PC3 cells contained more label into choline phospholipids than into Cho metabolites (Figure 9A). This was the opposite in PNT2C2 cells where the label detected in Cho metabolites was higher than in Cho phospholipids. These results also confirm the uptake data (Figure 8), indicating that PNT2C2 cells incorporate more label than the other cell lines under similar conditions. After labelling, most radioactivity was detected in PCho in all cell lines, especially in PNT2C2, LNCaP and PC3 lines; the least label was detected in Cho. Surprisingly, quite a high proportion of label was in GPCho, especially in P4E6 cells.

### Chronic phorbol ester treatment of PNT2C2 and PNT1A cells downregulates PKC*α* protein and reduces choline headgroup release

**Figure 10:**
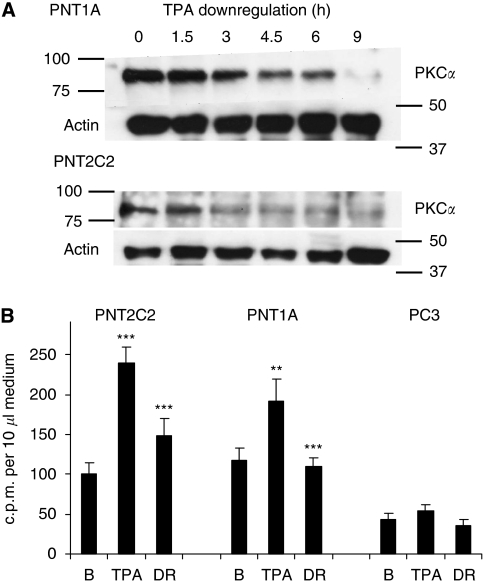
(**A**) Chronic treatment of PNT1A and PNT2C2 line cells with TPA downregulates PKC*α*. A total of 7.5 × 10^4^ cells were seeded into wells of 24-well plates, cultured for 24 h and treated with 250 nM TPA for the times shown, up to 9 h. Protein kinase C alpha was then resolved as described in Materials and Methods section. Blots were stripped and reprobed for actin as a loading control. Positions of 100, 75, 50 and 37 kDa markers are shown. (**B**) Reducing PKC*α* protein in PNT2C2 and PNT1A cells decreases the release of Cho metabolites into the medium. Cells were cultured and labelled with [^3^H]-Cho as in Methods. Cells were treated for 3 h with 4*α*-phorbol (basal, **B**), with 1 *μ*M TPA (TPA) or with 1 *μ*M TPA following downregulation of PKC*α* by treatment with 250 nM TPA for 9 h (DR). Results are mean c.p.m. per 10 *μ*l medium±s.d., *n*=4. As usual, PC3 line cells showed no significant TPA-stimulated release of choline headgroups. For PNT2C2 cells, TPA ^***^P<0.0001 against the basal value, for DR ^***^P<0.0001 against TPA value. For PNT1A cells ^**^P<0.001 against basal, ^***^P<0.0001 against TPA value.

Chronic exposure of PNT2C2 and PNT1A cells to 250 nM TPA for 9 h markedly reduced the PKC*α* protein content of cells (Figure 10A). Reprobing for actin indicated that approximately equal levels of total cell protein had been resolved, in agreement with previous results. TPA activation of PKC stimulated Cho metabolite release into the medium from both PNT2C2 and PNT1A lines. This effect was reduced to basal levels in PKC*α*-depleted PNT1A cells and by about 60% in PKC*α*-depleted PNT2C2 cells (Figure 10B). In contrast, TPA treatment of PC3 cells failed to stimulate significant Cho metabolite release over basal levels (Figure 10B), showing the variable effect of TPA on Cho metabolite release from PC3 cells.

## Discussion

### Protein kinase C alpha expression

We studied five cell lines to span the non-tumourigenic to metastatic extremes of PCa. PNT2C2- and PNT1A-immortalised cell lines were derived from normal prostate epithelia (Cussenot *et al*, 1991; Berthon *et al*, 1995). The P4E6-immortalised line was derived from an early prostate tumour (Maitland *et al*, 2001). The widely studied tumourigenic LNCaP and PC3 cell lines differ in their apoptotic response to PKC*α* activation, formation of metastases and regulation of the PI3K–PKB pathway (Sharrard and Maitland, 2007). We focused on PKC*α* because it regulates PLD (Cockcroft, 2001), which is linked to tumourigenesis (Foster, 2009). When normalised to GAPDH, PKC*α* protein expression varied considerably between the five cell lines, being weakest in P4E6 cells derived from an early prostate tumour (Figure 1D). This was surprising as PKC*α* expression is reportedly increased in PCa (Cornford *et al*, 1999; Koren *et al*, 2004; Lahn *et al*, 2004) and, in agreement, was 2–4 times higher in tumourigenic LNCaP and PC3 cell lines compared with P4E6 cells. This observation with P4E6 cells derived from an early tumour may indicate that PKC*α* protein expression is reduced in early PCa and that expression increases in later metastatic disease. However, the immortalised PNT2C2 and PNT1A cell lines from normal prostate epithelia express PKC*α* protein at the same level as the tumourigenic LNCaP and PC3 cell lines. A study of PKC*α* expression in primary prostate epithelial cells from normal and tumour tissue is in progress to determine whether immortalisation influences PKC*α* expression.

### Protein kinase C alpha signalling to MARCKS

Protein kinase C alpha regulates cell spreading and motility through the F-actin-binding protein MARCKS (Uberall *et al*, 1997; Larsson, 2006). Protein kinase C activation stimulates MARCKS phosphorylation in PNT1A, LNCaP and P4E6 cells (Figure 2), indicating that a phorbol ester-PKC*α*-MARCKS pathway is active even in the P4E6 line with its weaker PKC*α* protein content. Surprisingly, some MARCKS was constitutively phosphorylated in unstimulated PNT2C2 and PC3 cells. Novel PKC*ε*, a MARCKS kinase in fibroblasts (Uberall *et al*, 1997; Rombouts *et al*, 2008), might contribute to MARCKS phosphorylation in these prostate cell lines. However, PKC*α* protein turns over much more rapidly than PKC*ε* on chronic exposure of cells to phorbol ester (Olivier and Parker, 1992) and is barely detectable in PNT1A and PNT2C2 cells exposed to TPA for 9 h (Figure 10A). Myristoylated alanine-rich C kinase phosphorylation in prostate cell lines depleted in PKC*α* protein was reduced, indicating that PKC*α* is the major link to MARCKS. Phosphorylation of MARCKS causes its release from the plasma membrane exposing PI(4,5)P2 and regulating local F-actin organisation for cell spreading and focal adhesion formation (Larsson, 2006). Thus, constitutively phosphorylated MARCKS in PC3 cells could contribute to the increased motility and invasiveness shown by this line compared with LNCaP cells (Lang *et al*, 2002). MicroRNA-21 (miR-21), which is overexpressed in PCa (Krichevsky and Gabriely, 2009), targets MARCKS, promoting resistance to apoptosis and increased invasiveness (Li *et al*, 2009). MicroRNA-21 expression is higher in PC3 cells than in the LNCaP line (Li *et al*, 2009) contributing to their greater invasiveness.

### Phospholipase D activation by PKC*α*

Phospholipase D activity is elevated in many cancers and transformed cell lines (Foster and Xu, 2003; Foster, 2006); thus, we were surprised that levels of basal and PKC-stimulated PLD activity (Figure 3) were similar between the five cell lines. Protein kinase C alpha is specifically linked to activation of PLD1 (Kim *et al*, 1999; Cockcroft, 2001) and PLD2 (Chen and Exton, 2004); results show that a PKC*α* link to PLD is active in cell lines derived from both normal and tumourigenic epithelia, including P4E6 cells derived from an early tumour. Activation of PKC*α* increases PLD activity more in PC3 cells probably because this cell line expresses PKC*α*, PLD 1 and PLD2 prominently. Standard PLD assay conditions with 30 mM 1-butanol can interfere with the interaction between PKC*α* and PLD1, leading to a reduction in measured PLD1 activity (Hu and Exton, 2005) This might account for why basal PLD activity is similar in normal and tumourigenic cell lines (Figure 3). Myristoylated alanine-rich C kinase, which functions as a reversible source of plasma membrane PI(4, 5)P2 (Larsson, 2006), is a key regulator of the PKC*α*–PLD pathway (Sundaram *et al*, 2004).

### [^3^H]-label release

The centrifugation results (Figure 4) indicate that [^3^H]-label is not released into the medium from cell lines in any membrane-bound prostasome or exosome form (Whiteside, 2005; Nilsson *et al*, 2009). Differences in levels of basal [^3^H]-label release from the five cell lines (Figure 5A–E) must reflect a variation in the initial [^3^H]-Cho uptake into cells by Cho transporters (Michel *et al*, 2006), as well as in Cho metabolism and rates of PtdCho synthesis and turnover. Our uptake results (Figure 8) indicate that PNT2C2 cells, which release the highest levels of Cho metabolites, also show the greatest uptake of Cho over 60 min. The uptake results also confirm that LNCaP cells import Cho more rapidly than do PC3 cells, as noted by Baba *et al* (2007) and Muller *et al* (2009). Choline transporter expression has been partially defined for LNCaP and PC3 cells (Hara *et al*, 2006; Baba *et al*, 2007; Muller *et al*, 2009). According to Muller *et al* (2009), Cho uptake into LNCaP and PC3 cells involves a selective Cho transporter (Michel *et al*, 2006). Our finding that TPA-stimulated Cho metabolite release from PNT2C2 cells is not HC-3 sensitive suggests that a CTL1 family member (Michel *et al*, 2006) is not involved in the release mechanism. Choline metabolite release from PNT1A cells is partially HC-3 sensitive, suggesting a contribution by a CTL1-type component (Figure 6B). Bakovic (personal communication) comments ‘CTL1 could efflux free Cho as it regulates an ATP-independent, passive transport depending on Cho concentration gradient and that CTL1, as well as OCTs are probably not involved in PCho and GPCho transport, though the efflux of such metabolites has not been tested’ (Michel *et al*, 2006). Our results in Figure 9B indicate that most Cho taken up by all the cell lines is converted into PCho by CK as found by Hara *et al* (2006) for PC3 cells. After labelling, only non-tumourigenic PNT2C2 cells had label preferentially in water-soluble Cho metabolites (mainly PCho, Figure 9B) compared with phospholipids, perhaps indicating slower membrane turnover compared with tumourigenic cell lines. Phosphocholine levels in LNCaP and PC3 cells have been measured at about 0.8 and 1.2 mM, respectively, compared with 0.1 mM for senescent normal prostate epithelial cells (Ackerstaff *et al*, 2001; Glunde *et al*, 2006).

### Stimulated Cho metabolite release

Unstimulated prostate cell lines release GPCho, PCho and Cho into the medium in varying proportions. However, the main Cho metabolite released changes from PCho to Cho with the transition to tumourigenesis (Figure 7). Significantly, tumourigenic cell lines fail to show a consistent PKC-stimulated release of Cho metabolites (Figures 5A–E, 6A and B) compared with the marked stimulation shown by PNT2C2 cells derived from normal epithelia. PNT1A cells, also from normal prostate epithelia, occupy an intermediate position in that PCho and Cho are released in about equal proportions, whereas PKC activation stimulates Cho metabolite release more weakly than is detected with PNT2C2 cells. Other cell types, for example, fibroblasts, are known to release PCho into the medium on ATP stimulation (Chung *et al*, 1997). The TPA-stimulated release of Cho metabolites from PNT2C2 and PNT1A cells is reduced to basal levels by the widely used PKC inhibitors Ro31-8220 and GF109203X at 1 *μ*M concentration (Figure 6A). Neither inhibitor is specific for PKC; however, the MAPKAP kinase-1*β* and p70S6 kinase also inhibited by GF109203X and Ro31-8220 (Alessi, 1997) are not directly involved in PtdCho turnover and Cho metabolism. These inhibitor results indicate a PKC involvement in the TPA-stimulated release pathway and are also in agreement with the observations in Figure 3 that Ro31-8220 inhibits TPA-stimulated PLD activity in all the cell lines. PNT lines depleted in PKC*α* protein (Figure 10A) show reduced Cho metabolite release on restimulation (Figure 10B), further supporting the PKC*α* link to PLD. Therefore, in all cell lines, a TPA–PKC*α*–PLD pathway stimulates turnover of PtdCho to generate PtdOH and Cho. However, PCho is the main metabolite released by PNT2C2 cells, and thus Cho formed by PLD action must be converted into PCho before release. PNT1A cells release both PCho and Cho, indicating that the two non-tumourigenic cell lines differ in Cho metabolism, Cho transporter expression and PtdCho turnover. Phosphocholine may be released as a secondary signal (Cuadrado *et al*, 1993; Chung *et al*, 1997; Kiss and Mukherjee, 1997).

### Phosphocholine release from PNT2C2 cells

**Figure 11:**
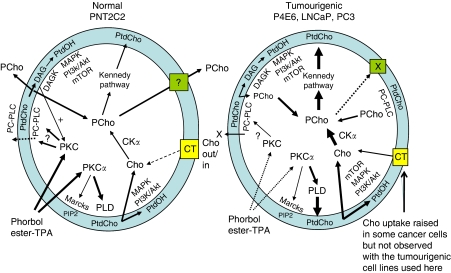
Summary of Cho metabolite formation and release from non-tumourigenic PNT2C2 and tumourigenic P4E6, LNCaP and PC3 cell lines. PNT2C2 cells: basal PC-PLC generates PCho, some of which is reused for PtdCho synthesis for membrane biogenesis and PtdCho turnover. DAG from PC-PLC activity might sustain long-term PKC activity and generates PtdOH via DAG kinase to regulate MAPK, mTOR, PI3K/Akt signalling for cell proliferation. Phosphocholine is released to function as an external secondary signal promoting growth factor signalling: PCho transporters are not identified. Phosphocholine may be released into the medium directly by PC-PLC translocated to the external cell surface. Protein kinase C isoforms may regulate PC-PLC translocation, which would increase on addition of phorbol ester. Protein kinase C activation by phorbol ester (TPA) stimulates PLD, increasing PCho formation, and may upregulate PC-PLC activity. In tumourigenic cell lines, PLD and CK*α* activities are upregulated to maintain high PCho levels for PtdCho formation for increased membrane biogenesis and PtdCho turnover for PtdOH formation. PtdOH promotes cell proliferation and malignant cell survival through MAPK, PI3K/Akt and mTOR pathways. PC-PLC activity is upregulated, increasing PCho formation. Increased Cho uptake as reported in some malignant cells (but not observed in these cell lines) would further increase PCho levels. Mechanisms of PCho release are downregulated to maintain high intracellular PCho levels in cancer cells. Phorbol ester (TPA) activation of PKC does not stimulate Cho formation by PLD and its release into the medium as it is rapidly phosphorylated to PCho by CK*α*.

In HeLa cells, basal turnover of PtdCho occurs through phosphatidylcholine-specific phospholipase C (PC-PLC), DAG kinase and lipid phosphate phosphatases, and does not involve PLD (Hii *et al*, 1991). Therefore, PCho released by PNT cell lines could be formed by PC-PLC activity, as is observed in normal and ovarian epithelial cancer cells (Spadaro *et al*, 2008) or in phorbol ester- or PDGF-stimulated fibroblasts (Podo *et al*, 1996; van Dijk *et al*, 1997). Involvement of a PC-PLC would explain the inhibition of Cho metabolite release from PNT lines by 100 *μ*M D609 (Figure 6B), initially reported as a PC-PLC inhibitor (Muller-Decker, 1989). However, D609 can inhibit PLD and a group IV PLA2 (Kiss and Tomoro, 1995; van Dijk *et al*, 1997; Kang *et al*, 2008), as well as sphingomyelin synthase (Luberto and Hannun, 1998). In epithelial ovarian cancer cells and NK cells, D609 has no effect on PLD or sphingomyelin synthase (Cecchetti *et al*, 2007; Spadaro *et al*, 2008). Intriguingly, PCho could also be released by the two PNT cell lines following translocation of a PC-PLC enzyme to the external surface of the plasma membrane (Ramoni *et al*, 2001, 2004). At this site, hydrolysis of PtdCho in the outer lipid leaflet would result in a direct release of PCho into the medium (Figure 11). As an example, exogenous *B. cereus* PC-PLC hydrolyses PtdCho in the outer lipid leaflet of fibroblasts (van Dijk *et al*, 1997). Such a translocation of PC-PLC may be regulated by PKC (Figure 11) as TPA can stimulate PC-PLC movement to the plasma membrane in fibroblasts (Ramoni *et al*, 2004). Hemicholinium 3 at 200 *μ*M, an inhibitor of CK (Jimenez *et al*, 1995) and of high- and medium-affinity Cho transporters (Michel *et al*, 2006), has no effect on TPA-stimulated Cho metabolite release from PNT2C2 cells. This supports the possibility that PC-PLC translocated to the cell surface releases PCho directly into the medium (Figure 11). Hemicholinium 3 partially inhibits PCho/Cho release from TPA-stimulated PNT1A cells, suggesting that Cho release via a CTL1 family transporter is blocked, whereas PCho release is unaffected.

### Choline metabolite release from tumourigenic cell lines

Our results show that, although PKC stimulates PLD activity in P4E6, LNCaP and PC3 cell lines (Figure 3), there is no consistent increase in Cho metabolite release into the medium (Figures 5C–E and 9). If the new Cho formed is not released, it must be rapidly converted into PCho by CK as detected in PC3 and LNCaP cells (Ackerstaff *et al*, 2001; Glunde *et al*, 2006; Hara *et al*, 2006) and in other malignant cells and cancers (Glunde *et al*, 2006). Choline kinase activity is upregulated in tumour-derived cell lines (Ramirez de Molina *et al*, 2002), probably accounting for the rapid conversion of Cho to PCho in tumourigenic prostate lines and why Cho is not released on PLD activation. Phospholipase D expression and activity is also increased in several cancers and malignant cell lines (Foster and Xu, 2003), which could further increase Cho formation. Such an increase was not detected in these tumourigenic prostate cell lines perhaps because of the butanol inhibition effects discussed above. Choline transport into tumourigenic cells may be increased (Figure 11), as has been observed in several cancer cell lines (Katz-Brull *et al*, 2002; Yoshimoto *et al*, 2004; Iorio *et al*, 2005), although we did not observe this with the cell lines studied here (Figure 8). Protein kinase C may also influence CK activity directly (Macara, 1989; Choi *et al*, 2005), further explaining why Cho is not released into the medium on TPA activation of PKC. PC-PLC activity may also be upregulated in tumourigenic prostate lines (Figure 11), as detected in ovarian and breast cancer cells (Glunde *et al*, 2004; Iorio *et al*, 2005, 2010), further increasing PCho formation. Elevated levels of PCho in neoplastic cells promote growth factor-induced mitogenic signalling to Raf-1 and MAP kinases (Cuadrado *et al*, 1993; Jimenez *et al*, 1995; Yalcin *et al*, 2010) and will also maintain flow through the Kennedy pathway, increasing PtdCho synthesis for membrane biogenesis (Figure 11) and cell proliferation, as well as to compensate for endosome formation for growth factor signalling (Vieira *et al*, 1996; Li *et al*, 1997). The *α* isoform of CK also affects cell-cycle regulation promoting both cell survival and proliferation (Ramirez de Molina *et al*, 2008; Chua *et al*, 2009). PtdOH generated in transformed cells also regulates cell proliferation and survival pathways via mTOR and Raf (Foster and Xu, 2003; Foster, 2007a, 2007b; Ramirez de Molina *et al*, 2008; Foster, 2009). An increase in cytidylyltransferase (CT) activity in tumourigenic prostate cells, as has been detected in some breast cancer lines (Eliyahu *et al*, 2007), would further increase PCho utilisation for PtdCho synthesis. A coupling between CT and PLD turnover of PtdCho, which might further stimulate PtdCho synthesis, has been reviewed (Cornell and Northwood, 2000). As PCho levels are elevated in neoplastic cells and transformed cell lines, it is relevant to ask why this metabolite is not released into the medium from tumourigenic prostate epithelial cells as occurs with normal PNT cell lines. Our findings with these cell lines infer that tumourigenesis in prostate epithelia results in the downregulation of normal mechanisms of PCho release into the medium so that high *intracellular* levels of PCho are maintained to enhance mitogen pathway signalling and PtdCho synthesis for increased cell proliferation and survival, as summarised in Figure 11.
